# HOUSING COSTS BURDEN AND SELF-RATED HEALTH: PRELIMINARY FINDINGS FROM A U.S. SAMPLE

**DOI:** 10.1093/geroni/igz038.3412

**Published:** 2019-11-08

**Authors:** Ji Hyang Cheon, John Cagle, Amanda Lehning

**Affiliations:** University of Maryland, Baltimore, Maryland, United States

## Abstract

Self-rated health is a multidimensional construct that includes not only physical health but also emotional and social well-being. Previous research has demonstrated that multiple factors contribute to individual self-rated health, including income. Because income is a somewhat limited indicator of older adults' financial circumstances, alternative measures such as housing cost burden may enhance our understanding of contributors to self-rated health. Further, because homeowners and renters may have a different attachment to their home and neighborhood, homeownership may moderate the association between housing cost burden and self-rated health. This study examined these relationships using data from 3,212 older adults in round 7 (2017) of the National Health & Aging Trends Study. Findings from multiple linear regression models indicate that the housing cost burden is associated with lower self-rated health, and this association is stronger for renters compared to homeowners. The findings indicate the potential for reduced housing cost burden to have a positive effect on health. The poster will conclude with practice and policy implications, including the potential benefits of expanding rental assistance programs to older adults who may not meet current income requirements but are experiencing high housing cost burden, as well as research implications, including the need for longitudinal approaches.

